# Are Human Dendrites Different?

**DOI:** 10.1016/j.tics.2020.03.002

**Published:** 2020-06

**Authors:** Mehmet Fişek, Michael Häusser

**Affiliations:** 1 Wolfson Institute for Biomedical Research, University College London, Gower Street, London, WC1E 6BT, UK; 2 Department of Neuroscience, Physiology, and Pharmacology, University College London, Gower Street, London, WC1E 6BT, UK

**Keywords:** dendrite, cortex, pyramidal cell, patch clamp, synaptic integration, neural computation, human, rodent

## Abstract

The first patch-clamp recordings from the dendrites of human neocortical neurons have recently been reported by Beaulieu-Laroche *et al**.* and Gidon *et al*. These studies have shown that human dendrites are electrically excitable, exhibiting backpropagating action potentials and fast dendritic calcium spikes. This new frontier highlights the potential for interspecies differences in the biophysics of dendritic computation.

Much of our understanding of how single neurons work has come from the study of model systems that have experimental advantages. Experiments on neurons of rats and cats, leeches and lobsters, squids and slugs, among others, have provided answers to fundamental questions about the molecular and cellular toolkit that is harnessed for computation. Many questions general to all nervous systems can be addressed this way, but certain mysteries specific to humans cannot be addressed with model systems. These include questions of obvious translational importance, such as: what are the pharmacological properties of human neurotransmitter receptors? Direct measurement of such properties can be crucial for developing new drugs for treating disease. However, beyond the clinical relevance of studying human neurons, such experiments form an essential part of our longstanding efforts to understand human cognition. Our remarkable cognitive abilities may arise from adaptations at both the cellular and network levels, and may depend, in some cases, on specific biophysical features that represent function-limiting constraints.

Dendrites represent most of the surface area of neurons and exhibit many properties that may form such function-limiting constraints. Most synapses are made onto dendrites and, thus, their passive and active properties determine how synaptic inputs are integrated before they influence the action potential output of neurons. Dendritic specializations can make the neuronal input–output function either linear and simple or richly nonlinear and complex [1]. Therefore, any specialization of human dendrites could impact the computations carried out by neurons and neural circuits and contribute to the cognitive specialization of humans. Moreover, the fact that the mammalian brain contains vast numbers of neurons – and dendrites! – means that even relatively small, quantitative differences in dendritic function could translate into significant computational advantages for human brains.

There is one obvious way in which human neurons and their dendrites are different from dendrites in, say, cats or rats: they are bigger. However, are these anatomical differences, which alone could change the signal processing capabilities of dendrites [2,3], also accompanied by differences in functional properties? Early intracellular recordings from neurons in resected cortical tissue from neurosurgical procedures (e.g., to treat epilepsy or brain cancer) revealed that human neurons appear to function largely similarly to those of other mammals [4]. However, more recent work using patch-clamp recordings has begun to reveal intriguing differences. For example, a single action potential can trigger a cascade of local network activity in human cortical tissue that lasts longer than in rodent cortex [5], and human layer 2/3 neurons appear to have a lower specific membrane capacitance compared with rodent neurons [6]. However, whether human *dendrites* also exhibit functional differences has remained unclear.

Now, two groups have made an important breakthrough: the first direct patch-clamp recordings from the dendrites of human pyramidal neurons in brain slices, which have revealed the functional properties of human dendrites. Beaulieu-Laroche and colleagues [7] reported that the dendrites of human cortical layer 5 pyramidal neurons have a similar repertoire of dendritic excitability to rat layer 5 neurons, but are less excitable, and that the additional length of human dendrites alters the input–output properties of the somatic and dendritic compartments. Specifically, the increased electrical isolation of apical dendrites in human layer 5 neurons could enhance the independence of computations in these remote dendritic regions, potentially providing a richer computational repertoire [1].

By contrast to the work of Beaulieu-Laroche on layer 5 dendrites, Gidon *et al.* [8] found that layer 2/3 dendrites are more excitable than rodent layer 2/3 dendrites: current injection into the dendrites of these neurons generated repetitive trains of fast dendritic calcium spikes, which can be independent of somatic action potentials. Dendritic spikes such as these are thought to be important for moment-to-moment computations as well as for triggering synaptic plasticity [1]. The calcium spikes found by Gidon *et al.* resemble calcium spikes in rodent cerebellar neurons [9] and dendritic spikes in rodent layer 2/3 neurons [10] but exhibit some key differences. Intriguingly, the authors show that these events show a paradoxical biphasic sensitivity to the magnitude of injected currents, reducing in amplitude with higher levels of stimulation. Building on previous work showing that dendrites can implement logical operations [11], the authors present a simplified model showing that dendrites that exhibit such sensitivity to input strength can, in principle, implement an exclusive-or (XOR) operation, where dendritic events would selectively amplify low levels of input, but not higher levels of input.

These ground-breaking studies have revealed that human cortical dendrites are electrically excitable and exhibit many similarities with dendrites in other species. However, they have also highlighted some tantalizing differences, inspiring many new questions. First, are the functional differences between human and rodent dendrites qualitative or quantitative? In other words, do they feature biophysically distinct dendritic events, or do they represent different points along a continuum? This requires careful side-by-side comparative studies across species, using identical protocols. The rarity of human tissue samples makes it difficult to control for their diversity: differences in disease states, brain areas, cell types, pharmacological history, and even the age and cognitive condition of the patient must be considered. Generalization across different neuron types in the human cortex is already proving difficult: there are some notable apparent discrepancies across recent studies, such as in specific membrane capacitance ([6] vs [7]) and dendritic excitability ([7] vs [8]), which will need to be carefully verified and explored further. Second, are the observed differences hallmarks of ‘specializations’ that are unique to individual species, or are they examples of physiological mechanisms that are variably found across the animal kingdom? Understanding such larger rules of organization will require careful comparisons across many species. Third, what are the biophysical origins of the observed functional differences, and how do they relate to molecular and anatomical differences between human and non-human neurons? This will require a systematic battery of experiments at many different levels, including transcriptional and pharmacological analysis (Box 1). Finally, how are the computational properties of human dendrites harnessed in the intact brain? This will ultimately require *in vivo* recordings from human dendrites (which face considerable practical and ethical challenges), complemented by simulations in large-scale networks of neurons with realistic dendritic properties. Answering these questions will allow us to identify precisely how our dendrites, and our brains, differ from those of other species, and also where they are the same, governed by common principles. Both outcomes would represent valuable contributions to our understanding of human cognition.

Box 1 **A Checklist for Cross-Species Comparisons of Dendritic Properties**

A systematic comparison of human and non-human dendrites would be immensely valuable, not only for probing and treating disease, but also for understanding the extent to which information processing in human dendrites might be distinctive, or instead reflects general principles across the animal kingdom. Where experiments in humans are challenging (e.g., *in vivo* measurements), recordings from non-human primates (particularly macaques and marmosets) will also be highly informative.

Here, we provide a ‘checklist’ for information that would permit a rigorous cross-species comparison:

• Morphological characterization of dendritic structure
• Transcriptome of human and non-human neurons
• Distributions of channels and receptors in dendrites
• Dendritic localization and trafficking of mRNAs
• Functional properties of dendritic excitability:
  - Passive electrotonic properties
  - Spread of backpropagating action potentials
  - Prevalence and types of dendritic spikes
  - Integration of patterns of synaptic inputs
  - Calcium and second messenger signaling pathways in dendrites
  - Synaptic plasticity mechanisms
• *In vivo* measurements of dendritic signals during behavior. Such experiments, yet to be carried out *in vivo* in humans, should explore how dendritic excitability is engaged by behaviorally relevant population activity, ideally during complex cognitive tasks.

Ultimately, this information must be integrated into mathematical models of neurons and networks, so that computational principles arising from the experimental findings can be rigorously explored. Embedding the results within a quantitative framework will also allow us to examine whether any observed differences between human and non-human dendrites lie along a continuum or represent a distinct feature, and also provide insights into the evolutionary path that has been taken to arrive at a particular feature set.

Alt-text: Box 1
